# Supplementary material for: Resolving the full spectrum of human genome variation using Linked-Reads
Source: Genome Res. 2019 Apr;29(4):635–45. doi: 10.1101/gr.234443.118 (PMC6442396; doi:10.1101/gr.234443.118)
Supplement: Supplemental Material [file supp_gr.234443.118_Supplemental_longranger-2.2.zip › longranger-2.2.tar.bz2!/lr2.2-public/lib/stan/cmdstan/stan/doc/api/html/math_2functions_2value__of_8hpp_source.html]

Stan: src/stan/math/functions/value\_of.hpp Source File


|  |  |
| --- | --- |
|  | Stan  2.5.0  probability, sampling & optimization |


- Main Page
- Related Pages
- Namespaces
- Classes
- Files

- File List
- File Members

All Classes Namespaces Files Functions Variables Typedefs Enumerations Enumerator Friends Macros Pages

- src
- stan
- math
- functions

value\_of.hpp

Go to the documentation of this file.

1 #ifndef STAN\_\_MATH\_\_FUNCTIONS\_\_VALUE\_OF\_HPP

2 #define STAN\_\_MATH\_\_FUNCTIONS\_\_VALUE\_OF\_HPP

3

4 namespace stan {

5

6  namespace math {

7

23  template <typename T>

24  inline double value\_of(const T x) {

25  return static\_cast<double>(x);

26  }

27

39  template <>

40  inline double value\_of<double>(const double x) {

41  return x;

42  }

43

44  }

45 }

46

47 #endif

stan::math::value\_of

double value\_of(const T x)

Return the value of the specified scalar argument converted to a double value.

**Definition:** value\_of.hpp:24

stan::math::value\_of< double >

double value\_of< double >(const double x)

Return the specified argument.

**Definition:** value\_of.hpp:40


---

[ Stan Home Page ]

*© 2011–2014,
Stan Development Team.*
